# Transarterial embolization of acute non-neurologic bleeding using Ethylene Vynil Alcohol Copolymer: a single-Centre retrospective study

**DOI:** 10.1186/s42155-023-00347-0

**Published:** 2023-01-26

**Authors:** Paolo Rabuffi, Antonio Bruni, Enzo Maria Gabriele Antonuccio, Andrea Saraceni, Simone Vagnarelli

**Affiliations:** Unit of Interventional Radiology, Azienda Ospedaliera San Giovanni Addolorata, Via dell’Amba Aradam 9, 00184 Rome, Italy

**Keywords:** Acute arterial hemorrhage, Embolization, Ethylene vinyl alcohol copolymer, Onyx, EVOH

## Abstract

**Background:**

To evaluate feasibility, safety and effectiveness of transarterial embolization of acute non-neurologic hemorrhage with Ethylene Vynil Alcohol Copolymer (EVOH).

**Methods:**

Between January 2018 and June 2021, 211 patients (male 123, mean age 69.7 y + 17.9) who underwent transarterial embolization with Onyx™ for acute non-neurologic arterial bleeding were retrospectively reviewed. Most frequent etiology of bleeding was post-operative (89/211, 42.2%), trauma (62/211, 29.4%) and tumor (18/211, 8.5%). Technical success was defined as the angiographic evidence of target vessel complete occlusion. Clinical success was defined as resolution of bleeding. Any rebleeding within the primitive site, requiring a new intervention during the first 30-days following embolization, was considered a clinical failure. Occurrence of procedure-related complication and mortality within 30 days of the embolization were examined.

**Results:**

A total of 229 embolization procedures was performed in 211 pts.; technical success rate was 99.5% (210/211 pts). Clinical success rate was 94.3% (199/211 pts). In 11 patients (5.2%) a reintervention was needed because of a rebleeding occurring within the primitive site, whereas in five patients (2.4%) rebleeding occurred within a site different from the primitive. Factors more often associated with clinical failure were coagulopathy/ongoing anticoagulant therapy (5/11, 45.4%), and post-operative etiology (3/11, 27.3%). EVOH was used as the sole embolic agent in 214/229 procedures (93.4%), in association with coils in 11 cases (4.8%), and with microparticles in 4 cases (1.7%). In the present series, major complications occurred in 6 cases (2.8%): respectively, four cases (1.9%) of colonic ischemia and two groin hematomas (0.9%) with active extravasation were observed. 26 (12.3%) patients died during the follow-up.

**Conclusion:**

Embolization of acute arterial bleeding with EVOH as a first-line embolic agent is feasible, safe and effective.

## Introduction

Acute arterial hemorrhage requiring urgent treatment may be related to several conditions such as trauma, tumor or post-surgical complications (Powerski et al. 2018). In the last decades transcatheter embolization (TE) has been succesfully used to treat arterial bleeding, resulting from different etiologies, in various anatomic locations and it is nowadays proposed as a first-line treatment in the management of acute hemorrhage (Chen et al. 2009, Wang et al. 2015). During TE, the effectiveness of the desired hemostasis must be balanced to the risk of ischemia or rebleeding. Since there are no established guidelines, the choice of the embolic agent depends on multiple factors, such as the operator preferences, the diameter or tortuosity of the target vessel and the presence of a pseudoaneurysm rather than an active extravasation. Onyx™ (Medtronic; Irvine, CA) is a liquid embolic agent which has been first introduced for the treatment of cerebral vascular malformations, but, thanks to its peculiar characteristics, in the last two decades it has also been increasingly used in the peripheral vascular district (Bommart et al. 2012, Guimaraes et al. 2011, Saeed Kilani et al.^2015^). This retrospective study was designed to determine the feasibility, effectiveness and safety of TE with EVOH as a primary embolic agent for the treatment of acute non-neurologic hemorrhage.

## Methods

The study was approved by the local institutional review board. Informed consent was obtained before treatment from each patients. All bleeding diagnosis were confirmed by multidetector CT before patients underwent angiography. A database of patients suffering from peripheral acute arterial bleeding, who underwent TE in an emergency setting between January 2018 and June 2021, was retrospectively analyzed and 211 patients (male 123, female 88, 16–97 years old) treated with EVOH were found. The demographics of patients, etiology, anatomic location of hemorrhage and nomenclature of treated vessels are listed in Tables 1 and 2. Indication for embolization procedure was determined by an interdisciplinary team, including anesthesiologists, surgeons, and the emergency medical team, based on variuous factors and including the hemodinamic condition of the patient. Patients who underwent embolization in the present study were hemodynamically stable (158/229, 69%) or showed a condition of mild to moderate hypotension which was successfully managed with the use of vasoactive agents (71/229, 31%). Onyx™ is a gelling solution composed of ethylene-vinyl alcohol copolymer (EVOH) and DMSO solvent. In order to polymerize, EVOH needs to be injected after the dead space of the microcatheter has been filled with DMSO; Onyx™ is available in two formulations, Onyx™ 18 (6% EVOH) and Onyx™ 34 (8% EVOH, which has almost double viscosity), that differ in terms of EVOH concentration and distal penetration. TE was performed in all cases after detection of bleeding at angiography; no empirical embolization was performed in any patient. First, selective catheterization of the target artery was performed with traditional diagnostic angiographic catheter or guiding catheter, according to the vessel diameter. Subsequently, a dimethyl sulfoxide (DMSO) compatible microcatheter (Carnelian, Tokai, The Hague, Netherlands; Excelsior SL-10, Stryker Neurovascular, Fremont, CA, USA) was superselectively inserted and placed in the desired position. Since injection of DMSO is painful, all patients systematically were administered Fentanyl (100–150 mcg) 10 minutes before the embolization, plus Propofol 1 mg/kg by intravenous route. All procedures were performed in the presence of an anesthesiologist.

**Table 1:** Baseline characteristics

**Gender**	**Frequency**	**Percentage**
Male	123	58.29
Female	88	41.71
Total	211	100
**Age classes**		
16-|37	14	6.64
37-|57	35	16.59
57-|77	75	35.55
77-|97	87	41.23
Total	211	100
**Etiology**		
Bronchiectasis	1	0.47
Coagulopathy	4	1.9
Diverticular disease	8	3.79
Vascular malformation	12	5.69
Pancreatitis	5	2.37
Post-operative	89	42.18
Early	28	13.27
Late	61	28.9
Trauma	62	29.38
Penetrating	8	3.79
Blunt	54	25.59
Tumor	18	8.53
Gastro-duodenal ulcer	12	5.69
Total	211	100
**Anatomic district**		
Head & Neck	15	6.6
Musculoskeletal (MSK)	109	47.6
Gastroenteric	54	23.6
Splancnic	51	22.3
Total	229	100

**Table 2:** Treated vessels

Treated arteries	Frequency
external carotid artery	1
bronchial artery	1
femoral circumflex artery	5
iliac circumflex artery	7
colic artery	11
hemorrhoidal artery	8
hepatic artery	7
inferior epigastric artery	27
ascending pharingeal artery	1
profunda femoris artery branches	11
superficial femoral artery branches	1
left gastric artery	6
gastroduodenal artery	15
gastroepiploic artery	1
genicular artery	1
gluteal artery	13
ileocolic artery	1
iliolumbar artery	5
intercostal artery	7
peroneal artery	1
lingual artery	3
lumbar artery	14
internal mammary artery	4
internal maxillary artery	3
superior mesenteric artery	2
humeral artery branches	2
obturatory artery	8
pancreatica magna artery	1
pancreaticoduodenal artery	7
popliteal artery	1
prostatic artery	5
internal pudendal artery	12
segmental renal artery	12
scapular circumflex artery	2
sphenopalatin artery	4
sigmoid artery	1
splenic artery	2
adrenal artery	1
superior thyroid artery	2
uterine artery	2
vesical artery	1
costocervical trunk	2
**Total**	**221**

### Study endpoint and definitions

Bleeding was defined as the angiographic evidence of extravasation or staining of contrast media, or in case of a vascular injury such as a pseudoaneurysm, necessitating urgent treatment. Technical success was defined as the angiographic evidence of target vessels complete occlusion. Clinical success was defined as resolution of bleeding, without need of reintervention in the following 30 days. The parameters used to assess clinical success of embolization were the absence of further decrease in haemoglobin levels or new bleeding episodes after the procedure. Any rebleeding within the primitive site occurring during the first 30-days following embolization, and requiring a new intervention, was considered a clinical failure. Coagulopathy was defined in presence of one or more of the following: thrombocytopenia (platelet counts lower than 50,000/mm^3^), a prothrombin time lower than 50% of the coagulation activity of normal reference plasma or an activated partial thromboplastin time of 50 seconds or more. In all cases, coagulopathy was treated with platelets or fresh blood plasma infusion, or anticoagulants antidotes (such as vitamin K, or DOAC specific reversal agents) according to its etiology. Procedure-related complications were defined based on the European Society of Cardiovascular and Interventional Radiology (CIRSE) standard of practice committee classification system (Filippiadis et al. 2017). Follow-up was conducted for 30 days after the intervention, by retrospectively reviewing clinical data in our electronic medical record system and by telephone calls.

### Statistical analyses

Univariate and bivariate analyses were performed to determine both the clinical success and the predictive factors associated with it. The results of univariate analysis were tested for significance using hypothesis testing for proportion. The Chi-square test was used to determine whether two variables are likely to be related or not. For all statistical tests, *p*-values < 0.01 were considered significant. All statistical analyses were performed by using software R (version 4.2.).

## Results

A total of 229 embolization procedures was performed in 211 pts. TE with EVOH in the present series was technically successful in 99.5% of patients (210/211). Clinical success rate was 94.3% (199/211 pts). EVOH was used as the sole embolic agent in 214/229 procedures (93.4%), in association with coils in 11 cases (4.8%), and with microparticles in 4 cases (1.7%). EVOH 6% formulation was used exclusively in 168 cases (73.4%), EVOH 8% in 41 cases (17.9%); a combination of the two EVOH concentrations was injected in 20 cases (8.7%) (Table 3). The mean number of 1.5-ml vials of EVOH injected was 1.4 per procedure. 16 patients (7.6%) underwent a new embolization for a bleeding recurrence. In 11 patients (5.2%) a reintervention was needed because of a rebleeding occurring within the primitive site, whereas in five patients (2.4%) rebleeding occurred within a site different from the primitive. Characteristics of rebleeding cases are listed in Table 4. Factors more often associated with clinical failure were coagulopathy/ongoing anticoagulant therapy (5/11, 45.4%), and post-operative etiology (3/11, 27.3%). The EVOH 8% formulation was used in 72.7% of clinical failure cases and in 73.4% of the remaining general population, so there was no significative difference regarding EVOH formulation between clinical success and clinical failure cases. In the present series, major complications occurred in 6 cases (2.8%): respectively, four cases (1.9%) of colonic ischemia and two groin hematomas (0.9%) with active extravasation were observed. 26 patients (12.3%, mean age 74.1 y, 28–94) died during the follow-up. One patient, who underwent colectomy because of colonic ischemia following TE, died for multiorgan failure after 7 days. The other 25 patients died for the consequences of trauma (7/25), or because of the severity of their underlying disease (18/25).

**Table 3:** EVOH concentration, volume injected, use of other embolic agents in association

EVOH concentration	Frequency	Percentage
EVOH 8%	41	17,9
EVOH 6%	168	73,4
EVOH 6% + 8%	20	8,7
Total	229	100
**EVOH 6% 1.5 ml vials injected**		
1	125	74,4
2	33	19,6
3	4	2,4
4	5	3
5	1	0,6
Total	168	100
**EVOH 8% 1.5 ml vials injected**		
1	29	70,73
2	7	17,07
3	3	7,32
4	2	4,88
Total	41	100
**Other embolic agents**		
No	214	93,4
Coils	11	4,8
Microspheres	4	1,7
Total	229	100

**Table 4:** Rebleedings

Age, sex	Etiology	Timing (days)	Bleeding site	EVOH concentration	Injected volume (vials)	Other embolics
79y, F	pancreatitis	1	gastroduodenal	8%	1	No
86y, F	coagulopathy (OAT)	1	lumbar	6%	1	No
41y, M	trauma	1	pudendal	6%	1	No
62y, M	post-operative (robotic prostatectomy)	1	prostatic	6%	1	No
45y, F	post-operative femoral puncture	4	femoral circumflex	6%	1	No
85y, F	post-operative (femoral prosthesis)	15	iliac circumflex	6%	3	No
74y, M	larynx tumor	30	external carotid	8%	1	No
79y, F	coagulopathy (OAT)	1	inferior epigastric	6%	1	No
88y, M	coagulopathy (OAT)	1	iliolumbar	8%	1	No
75y, M	coagulopathy (OAT)	7	scapular	8%	1	No
50y, M	coagulopathy (cirrhosis)	12	intercostal	8%	1	No

*OAT* Oral Anticoagulant Therapy

## Discussion

Overall clinical success of TE with EVOH in the present series was 94.6%; the highest clinical success rate was observed within the subgroup of patients suffering from post-traumatic bleeding (98.3%), whereas the lowest efficacy rate was recorded within the subgroup of patient suffering from coagulopathy or under anticogulant therapy (91.9%). Clinical success rate among the patients suffering from upper and lower gastro-intestinal bleeding (UGIB and LGIB, 52/211, 24.6% of total cases) was 96.1%. The choice of the appropriate embolic agent is still a controversial matter. The ideal embolic agent should be safe, compatible with most microcatheters, easy to prepare and control during injection and able to achieve an efficient occlusion. Clinical efficay rate of coil embolization for the treatment of acute arterial bleeding is 76–90% (Tan et al.^2010^, Khanna et al. 2005, Valek et al. 2013, Yap et al. 2013, Kickuth et al. 2008). However, the use of coils is contraindicated in case of false aneurysms and may not be feasible within small, tortuous target arteries or in a vasospasm condition. Embolization of gastrointestinal bleeding with N-butyl-cyanoacrilates (NBCA) has proven to be effective, with clinical success rates ranging from 70.4%–74.5% to 78%–88% for, respectively, upper and lower gastro-intestinal bleeding (UGIB and LGIB) (Hur et al. 2014, Hur et al., 2017). However, obtaining an efficient distal distribution of NBCA requires great experience and technical skill (Hill et al. 2018): infact, since NBCA has a short polymerization time, once the mixture with Lipiodol is prepared, it must be injected immediately in order to avoid catheter lumen occlusion. Nevertheless, reflux of glue during injection may happen, with non-target embolization events reported in up to 16.7% of cases (Guimaraes et al. 2011, Mavili et al. 2007).

**Fig. 1:**
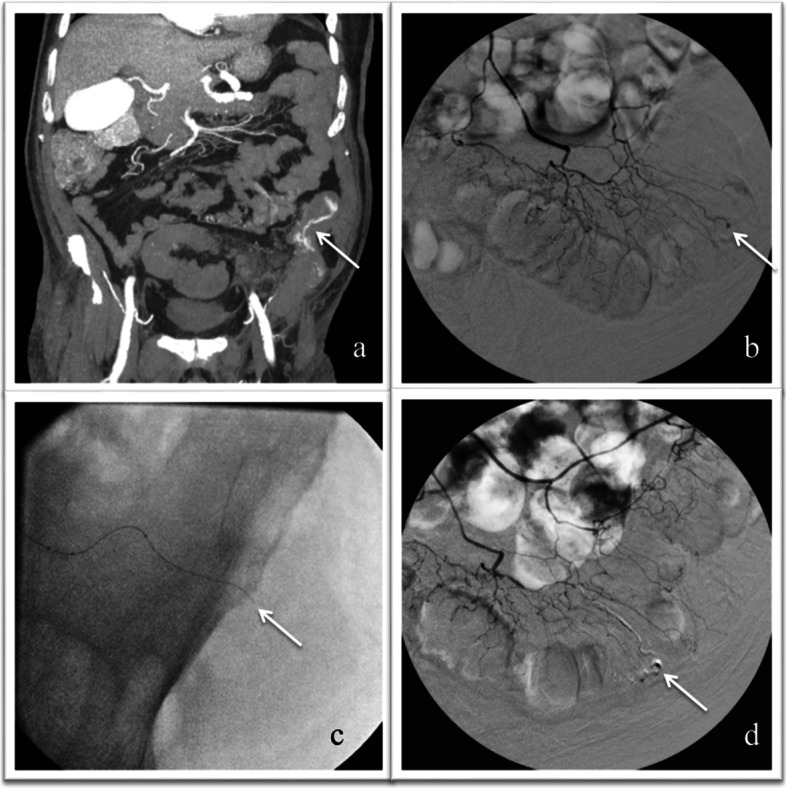
**a**–**d** CT MPR images in a patient suffering from lower gastrointestinal bleeding show active diverticular bleeding within the lumen of the descending colon (**a**). Selective inferior mesenteric artery angiography (IMA) shows a contrast extravasation from a branch of the sigmoid artery (**b**). After superselective catheterization of the target vessel with a 1.7 Fr microcatheter (**c**), selective IMA post-embolization angiography confirms selective occlusion of the target vessel (**d**)

**Fig. 2:**
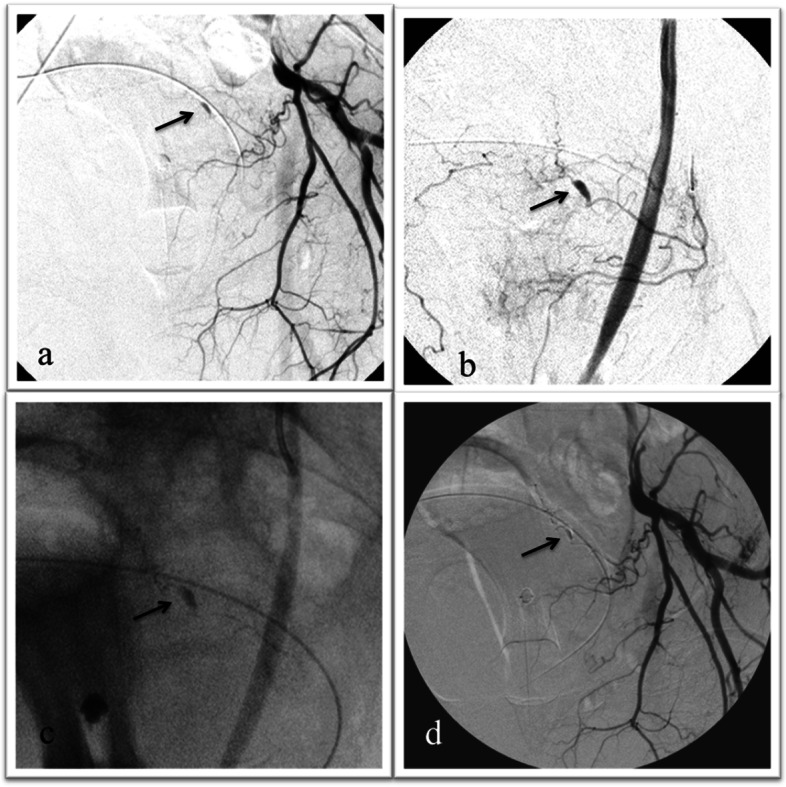
**a**–**d** Selective left internal iliac artery angiography (IIA) in a patient suffering from pain and anemia after robotic prostatectomy shows the presence of a false aneurysm of the distal branch of the superior vescical artery (**a**). After superselective catheterization of the target vessel with a microcatheter (**b**), embolization is performed with Onyx 18. Filling of the pseudoaneurysmal sac, distal and proximal portion of the vescical artery is visible in **c**. IIA post-embolization angiography confirms effective occlusion of the target vessel

The characteristics that made EVOH a popular embolic agent within the neurovascular system allow its use also for the embolization of peripheral vascular arteries. In fact, thanks to its 5 minutes-long polimerization time, high radiopacity and easy diffusion within tortuous and small-caliber vessels, EVOH has proven to be a safe and efficient embolic agent for the treatment of hemoptysis and traumatic hemorrhage, with clinical success rates of 94% and 100%, respectively (Ayx et al. 2017, Muller-Wille et al. 2012). High clinical success rates, ranging from 96.7% to 100%, were also reported using EVOH in the embolization of LGIB and renal acute arterial hemorrhage (Urbano et al. 2014, Lenhart et al. 2010, Mahdjoub et al. 2020). Compared to NBCA, EVOH injection can be stopped and resumed during the embolization, since the copolymer is nonadhesive and solidifies in an “outside-in” fashion, allowing the operator to perform longer injections with lesser ocurrance of microcatheter entrapment or occlusion (Guimaraes et al. 2011, Poursaid et al. 2016, Loh et al. 2010). In the present series 6% EVOH formulation was preferred in 73.4% of cases, because of its lower viscosity and more distal penetration capacity, in comparison to 8% EVOH (Figs. 1 and 2).

EVOH can be either used as a flow-dependent embolic agent, relying on arterial pressure-driven fluidodynamics in order to navigate within the target vessel, or, as it is done in case of cerebral AVMs, after balloon-occlusion of the artery from which the injection is performed, in order to neutralize the pressure gradient and achieve a more distal EVOH penetration. Another technique used to maximize distal penetration of EVOH is to navigate superselectively the target vessel until the microcatheter occupy entirely the arterial lumen: in such situation the vessel flow is blocked and the embolic agent may be pushed very distally facing low resistances during injection. In the present series no occurrance of rupture nor entrapment of the microcatheter tip were observed. Coils were used in combination with EVOH in 11 cases, in the present experience. The rationale of using EVOH in association with coils is to obtain a flow deceleration, expecially in case of large vessel with very high flow, in order to reduce the risk of non-target embolization. Also, in the present series, EVOH plus coil embolization was performed in case of target bleeding vessel too small to be catheterized (ie, small sidebranches of the gastroduodenal artery): in such cases, after the main trunk of the parent artery, distally to the bleeding sidebranches, was occluded with coils, EVOH was able to navigate up to the coil plug, and then, through a retrograde filling, embolize the target bleeding sidebranch. In this series, EVOH was used in combination with microspheres in 4 cases (100–300 and 300–500 μm in 2 embolization of hepatic arteries for ruptured HCC, and 500–700 μm in 2 embolizations of uterine arteries for bleeding cercival cancer). The rationale of using microspheres was to obtain, at first, an occlusion of the distal feeding vasculature of the lesions before completing embolization with EVOH, in order to reduce the risk of vessel recanalization and possible rebleeding in the following days. Another point in favor of embolization with EVOH is that the microcatheter is not subject to overload nor friction during EVOH inection and delivery, whereas dislocation of the microcatheter may happen during coil insertion and deployment due to the mechanical stress to which the device is subjected. Finally, compared to particles that require larger inner lumen microcatheter as the size of the used microsphere increases, 6% or 8% EVOH, despite their different viscosity, may be injected through a microcatheter of the same size. Recurrency of hemorrhage after TE with coils or gelfoam has showed, in recent publications, consistent rates at 30 days ranging from 23% to 27.8% for gastrointestinal bleeding and 20.8% for peripheral acute arterial bleeding (Yap et al. 2013, Sirvinskas et al. 2017, Beggs et al. 2014, Powerski et al. 2018). In comparison to traditional embolic agents, embolization with NBCA for non-variceal gastrointestinal bleeding has been associated with lower rebleeding rates, of, respectively, 12.5% and 15.7% for UGIB and LGIB (Chevallier et al. 2021). There is paucity of data regarding recurrence of bleeding after embolization with EVOH: in the only published meta-analysis available, a rebleeding rate of 7.6% was described, which was higher in case of hemoptysis, compared to gastrointestinal hemorrhage (Kolber et al. 2015). In his experience of LGIB embolization with EVOH, Urbano reported a recurrence of the bleeding in 10% of cases, though the relevance of the data is probably overestimated, since two-thirds of the rebleeding occurred in sites different from the primitive, and in no case a reintervention was needed (Urbano et al. 2014). In the present series 11 patients (mean age 69.4 years) needed a new embolization for a bleeding recurrence in the primitive site (5.4% of cases), after a median time of 6.7 days (1–30 days). Within this subgroup of patients, five (45.4%) were suffering from coagulopathy or under anticoagulant therapy and three patients (27.3%) underwent a surgical or endovascular intervention before (see Table 4). Only one patient out of 45 who underwent embolization with EVOH in the present case series for gastro-intestinal hemorrhage experienced a rebleeding within the first 30 days from the intervention. Coagulopathy represents an independent predictor for clinical failure and recurrency of bleeding after embolization (Loffroy et al. 2020, Kim et al. 2017). In case of coagulopathy, permanent liquid embolic agents such as EVOH and NBCA are preferred over coils or gelfoam because they act independently of the patient coagulation status (Hur et al. 2014, Hur et al., 2017). In fact, in this condition clinical failure rate of TE with non-liquid embolic agents has been reported to be as high as 45–64%, whereas embolization with NBCA showed a clinical failure rate of 33.8% (De Wispelaere et al. 2002, Defreyne et al. 2003, Loffroy et al. 2015). In the present series, the subgroup of patients in whom coagulopathy was the primary cause of bleeding accounted for 62 units (29.4% of total population): in those patients, embolization with EVOH showed a clinical success rate of 91.9% and a rebleeding rate of 8.1%, which is significantly lower than previously reported using traditional embolic agents or NBCA. Major complications following TE with coils and other non-liquid embolic agents range may occur in up to 23% (Maleux et al. 2009, Ahmed et al. 2010, Navuluri et al. 2012) of patients. The use of NBCA for embolization of non-variceal gastrointestinal bleeding has been associated with a major complication rate of 8.6% (Chevallier et al. 2021), whereas major complication rate after embolization with EVOH has been described as low as 3.1% (Kolber et al. 2015). In the present series 6 major complication were recorded (2.8%). Four patients experienced colonic ischemia after embolization with EVOH, due to unintended non-target vessel embolization: in one case a surgical colectomy was required, whereas in the other three patient the ischemia resolved spontaneously after conservative management. In two cases, following TE, a groin hematoma with active bleeding, requiring embolization was observed. In the present study, the rate of complication following TE ranged from 0% of post-traumatic hemorrhage, to 7.1% of external carotid branches embolization and 7.6% of gastrointestinal bleeding.

There are some aspects to take into consideration when using EVOH: DMSO must be injected slowly to prevent vasospasm and requires exclusive use of compatible catheters. Also, the solvent injection is painful, therefore the presence of an anesthesiologist is recommended in order to perform a proper analgesia. Moreover, to ensure radiopacity, the EVOH vials need to be shaken for at least 20 minutes prior to its use, hence the dedicated vial-mixers should be activated as soon as the patient is referred to the angio suite. Although embolization with EVOH requires a longer learning curve compared to coils or other traditional embolic agent, this disadvantage is overcome by the major hemostatic power of EVOH, which is not influenced by the coagulation status of the patient, and by the fact that EVOH is more controllable during injection with very low occurrance of non-target embolization.

### Limitations

The present study has some limitations: it is retrospective, has no control group, and reflects a single-institution experience.

## Conclusion

Embolization of acute arterial bleeding with EVOH has showed to be feasible, safe and effective, confirming the data reported from other authors who described similar patient series. Although the emergency setting of acute arterial bleeding makes it particularly difficult to set up a prospective randomized study comparing the various embolic agents, certainly further studies with larger populations are needed in order to confirm these results. (Figs. 1 and 2).

## Data Availability

All data and materials related to the study are available if requested.

## Abbreviations

EVOH: Ethylene Vynil Alcohol
TE: Transcathether Embolization
DMSO: Dymethil Sulfoxide
NBCA: N-Butyl-Cyanoacrilates
LGIB: Lower Gastrointestinal Bleeding
UGIB: Upper Gastrointestinal Bleeding
